# Accuracy assessment of measuring component position after total ankle arthroplasty using a conventional method

**DOI:** 10.1186/s13018-017-0611-2

**Published:** 2017-07-31

**Authors:** Kyoung-Jai Lee, Shao-Hua Wang, Gun-Woo Lee, Keun-Bae Lee

**Affiliations:** Department of Orthopaedic Surgery, Chonnam National University Medical School and Hospital, 42 Jebongro, Donggu, Gwangju, 61469 Republic of Korea

**Keywords:** Total ankle arthroplasty, Conventional method, Component position

## Abstract

**Background:**

This study was to assess the accuracy of measuring the tibial and talar components position and to investigate the outlier rate of each component and predisposing factors related to component malalignment after total ankle arthroplasty (TAA) using a conventional method.

**Methods:**

One hundred fifty consecutive primary total ankle arthroplasty were performed using the three-component HINTEGRA prosthesis for ankle end-stage osteoarthritis. Radiographic analysis for the accuracy of component position in coronal and sagittal plane was conducted at postoperative 6 months. Additionally, the accuracy of component position was evaluated according to presence of preoperative deformity or joint incongruency.

**Results:**

The mean postoperative coronal angles of the tibial and talar components (α and γ) were 91.9° and 91.3°. The mean postoperative sagittal angles of the tibial and talar components (β and δ angle) were 84.6° and 91.7°. In the coronal plane, 16 (10.7%) tibial components and 15 (10.0%) talar components showed outliers greater than 5°. In sagittal plane, 15 (10.0%) tibial components and 29 (19.3%) talar components showed outliers greater than 5°. There was no meaningful increase of the outlier rate regarding presence of preoperative deformity or joint incongruency.

**Conclusions:**

In conventional method of TAA, the outlier rate of the tibial and talar components was about 10 to 20%, especially, the outlier rate of talar component in sagittal plane was up to 20%. Therefore, careful attention should be paid to implant the talar component in conventional TAA.

## Background

Total ankle arthroplasty (TAA) has been widely used for the treatment of end-stage ankle arthritis in recent years and satisfactory results have been reported [1–6]. However, high complication and reoperation rates after TAA have also been reported and still remain unsolved [7–10].

The success of joint arthroplasty depends on many factors, including patient selection, prosthetic design, soft tissue balancing, severity of joint deformity, and component position [11–17]. Among these factors, proper component position is one of the most important factors, which influences the longevity of the implant and prevents complications. Survivorship was also typically diminished when optimal alignment is not achieved [18–23]. Therefore, ankle prostheses should be aligned in such a way to assure even distribution of forces on the polyethylene liner [24].

Previous studies have reported radiographic outcomes and predisposing factors that increase the risk of postoperative malalignment after hip or knee joint arthroplasty. The malalignment of component has been reported ranges from 4 to 23% after primary total hip arthroplasty. Severe varus deformity and retroversion of the femur can cause incorrect insertion of the stem [25, 26]. In total knee arthroplasty, severe preoperative varus or valgus deformity and femoral bowing are associated with increased risk of alignment problem. Then, the prevalence of malalignment in total knee arthroplasty has been reported range from 5 to 21% [27–29].

There are several studies about the accuracy assessment of component position following TAA. The rate of malalignment was varied from 0 to 35% after TAA. However, in these studies, the criteria for acceptable alignment of component was not standardized, and there are no comprehensive studies conducted regarding the outlier rates of the tibial and talar components or predisposing factors of incorrect component position following TAA. Therefore, the purpose of the present study was to evaluate the accuracy of component position after conventional TAA and to analyze the predisposing factors that affect outliers by using radiographic analysis.

## Methods

### Patient

This study was approved by our institutional review board, and informed consent was obtained from all patients. Between January 2005 and December 2011, 153 consecutive TAAs were performed by a single surgeon in 146 patients (153 ankles) with symptomatic end-stage ankle osteoarthritis using cementless mobile-bearing HINTEGRA prosthesis (Newdeal, SA, Lyon, France). Three patients (3 ankles) were excluded because plain radiographs were not available to measure radiographic parameter. The study cohort consisted of 150 ankles (93 men, 57 women) with a mean age of 61.6 years (42 to 82). Ninety (60%) ankles were diagnosed with posttraumatic ankle arthritis, and 60 (40%) ankles were diagnosed with primary ankle osteoarthritis.

### Operative technique and postoperative rehabilitation

All patients received total ankle arthroplasty by a single surgeon using a longitudinal anterior approach between the anterior tibial tendon and the extensor hallucis longus with the patient in the supine position. After removal of anterior capsular synovial tissue and osteophytes, the tibial cut was made perpendicular to the mechanical axis of tibia in coronal plane and had a posterior slope of 6° in sagittal plane while sparing as much subchondral bone as possible. The talar cut was made parallel to the tibial cut in coronal plane and parallel to the sole, then the medial, lateral, and finally posterior talar cuts were made. After the selected implants were inserted, the alignment, stability, and joint motion were checked clinically, while component position was checked by image intensification. The wound was closed in a standard fashion and a closed suction drain was inserted.

All patients followed the same postoperative protocol. In order to keep the foot in a neural position, a short leg splint and non-weight bearing were necessary for the first 2 weeks after surgery. For the next 4 weeks, patient was instructed to start gentle range of motion (ROM) exercise. Progressive weight-bearing ambulation was initiated with ankle-foot orthosis at 6 weeks after surgery. The full weight-bearing ambulation and rehabilitation program, which included stretching of the triceps surae, calf strengthening, and proprioceptive exercise were allowed to start at 8 to 10 weeks after the surgery. The period of immobilization may differ a little whether additional procedures were performed or not.

### Radiographic evaluations

Radiographic examinations including anteroposterior and lateral radiographs of the ankle taken preoperatively, immediate postoperatively, at 3 and 6 months postoperatively, and annually thereafter. With preoperative radiographic analysis, we conducted radiographic analysis after 6 months of operation when the patients were able to perform weight-bearing exercises. For the radiographic analysis, we measured the following by using PACS (Picture Archiving and Communication Systems: Marotech 5.4).

**Fig. 1:**
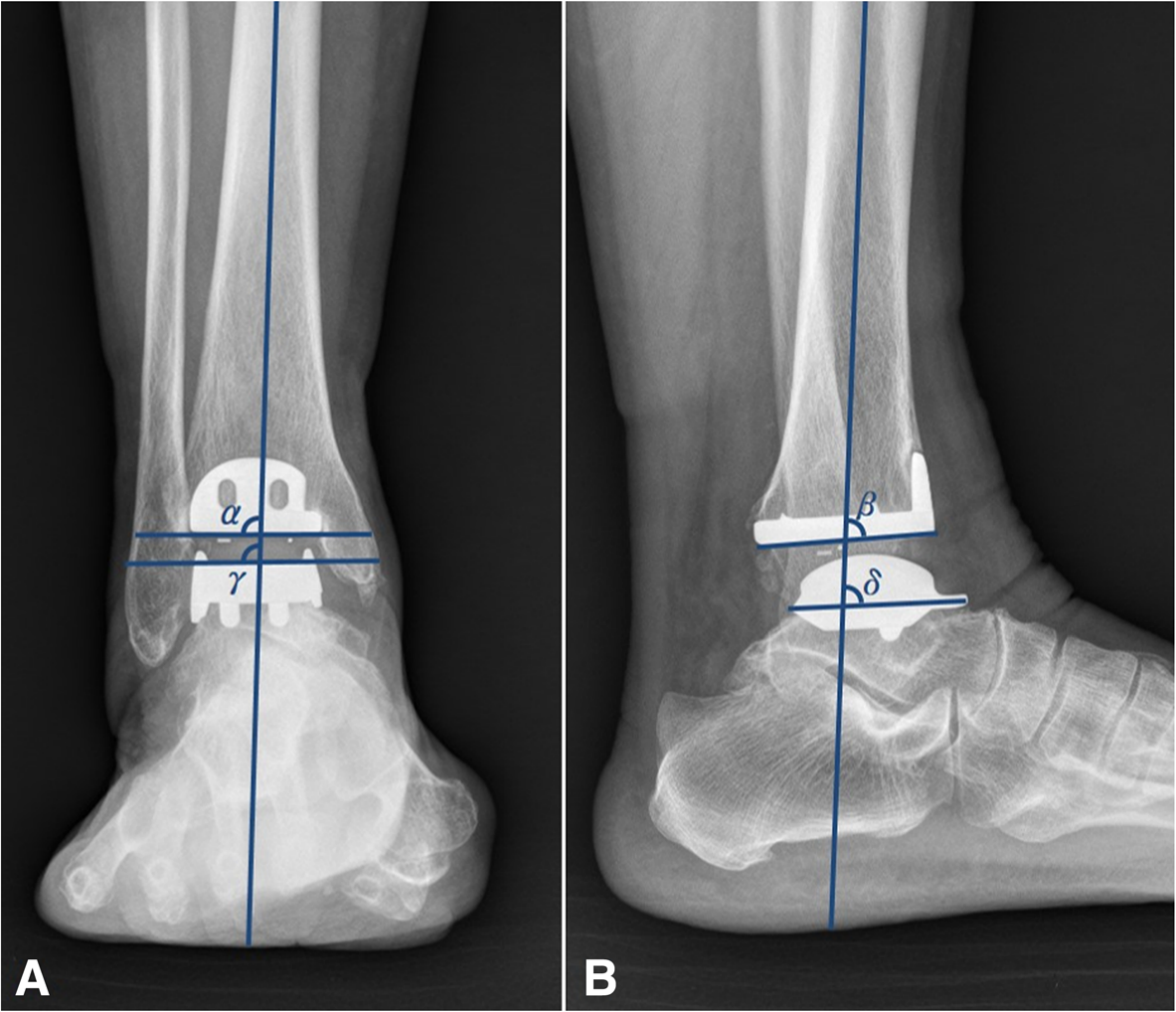
The measurement of the angular position of the component. **a** α and γ are the angles on the anteroposterior view between the longitudinal axis of the tibia and the articulating surface of the tibial and talar components. **b** β and δ are the angles on the lateral view between the longitudinal axis of the tibia and the articulating surface of the tibial and talar components

We evaluated the accuracy of component position depending on the type of preoperative coronal deformity: varus, neutral, or valgus. Neutral position in coronal plane was defined as within 5° of varus or valgus on anteroposterior radiograph. On preoperative radiograph, 80 (53.3%) ankles showed coronal varus tilting above 5°, 28 (18.7%) ankles showed valgus tilting above 5°, and 42 (28%) ankles showed coronal tilting below 5° of varus or valgus. Coronal positions of the tibial and talar components were assessed using α and γ angles (Fig. 1a).

As done with coronal plane analysis, we evaluated the accuracy of component position depending on preoperative sagittal deformity: extension, neutral, and plantar flexion. The aimed value of the anterior distal tibial angle was 84°. Therefore, we defined, a neutral position in sagittal plane as being in the rage of 79° to 89° of the anterior distal tibial angle. On the sagittal plane, 85 (56.7%) ankles showed extension of the distal tibial slope (anterior distal tibial angle <79°) above 5°, 7 (4.7%) ankles showed plantar flexion of the distal tibial slope (anterior distal tibial angle >84°) above 5°, and 58 (38.7%) ankles showed sagittal tilting below 5°. Sagittal angular positions of the tibial and talar components were assessed using β and δ angles on postoperative radiographs (Fig. 1b).

In addition, we evaluated the congruency of the ankle joint. An ankle was defined as congruent when the difference between the tibial and talar articular surface was less than 5°. Accordingly, four categories of deformity were defined: varus-congruent, varus-incongruent, valgus-congruent, and valgus-incongruent.

Outcomes were defined as “excellent” when the difference between the postoperative alignment and aimed value was within less than 3°, “acceptable” when more than 3° and less than 5°, and “outlier” when more than 5° from aimed value in the coronal-tibial(α), coronal-talar(γ), sagittal-tibia(β), and sagittal-talar(δ) angle.

To avoid potential bias, plain radiographs were evaluated by two independent observers who were not involved in the surgical treatment of the patients and who were blinded to the intention of this study.

### Statistical analysis

Descriptive statistics (arithmetic means, standard deviations, and ranges) were calculated with use of standard formulas. The one-way analysis of variance (ANOVA) test was used to analyze differences in continuous variables such as α, β, γ, and δ angles among three groups. Tukey’s honestly significant differences (HSD) was performed was used for post-hoc comparisons. The Fisher’s exact test was used to analyze differences in outcomes such as “excellent”, “acceptable,” and “outlier”. A *p* value of <0.05 was considered significant, and all aspects of the statistical analysis were reviewed by a statistician.

## Results

### Accuracy assessment of component position

The mean coronal angle of the tibial and talar components (α, γ angle) were 91.9° and 91.3° at 6 months postoperatively (Table 1). In terms of accuracy of component position, 86 (57.3%) and 90 (60.0%) of the tibial and talar components were excellent, and 48 (32.0%) and 45 (30.0%) were acceptable. There were 16 (10.7%) and 15 (10.0%) coronal outliers for the tibial and talar components.

**Table 1:** Accuracy of implant position after total ankle arthroplasty

	Coronal alignment		Sagittal alignment	
	α angle	γ angle	β angle	δ angle
Angle^a^	91.9 ± 2.7	91.3 ± 3.1	84.6 ± 3.0	91.7 ± 4.1
Outcomes^b^				
Excellent	86 (57.3%)	90 (60.0%)	98 (65.3%)	76 (50.7%)
Acceptable	48 (32.0%)	45 (30.0%)	37 (24.7%)	45 (30.0%)
Outlier	16 (10.7%)	15 (10.0%)	15 (10.0%)	29 (19.3%)

^a^The values are given as the mean and the standard deviation

^b^The values are given as the number of ankles with the percentage in parentheses

α and γ angles are measured on anteroposterior radiographs between the longitudinal axis of the tibia and the articulating surface of the tibial component or talar component

β and δ angles are measured on lateral radiographs between the longitudinal axis of the tibia and the articulating surface of the tibial component or talar component

The ideal values of α, γ, and δ angles are 90°, the ideal value of β angle is 84°. Outcomes were defined as “excellent” when values were within 3°, “acceptable” when within 5°, and as “outlier” when more than 5° from optimum values

The mean sagittal angle of the tibial and talar components (β and δ angle) were 84.6° and 91.7° at 6 months postoperatively (Table 1). Fifteen (10.0%) and 29 (19.3%) outliers for sagittal angle of the tibial and talar components were occurred. Ninety-eight (65.3%) and 76 (50.7%) of the tibial and talar components were excellent, and 37 (24.7%) and 45 (45.0%) were acceptable.

### Predisposing factors for outlier of component position

We evaluated the accuracy of implant position depending on preoperative coronal deformity: varus, neutral, and valgus (Table 2). In coronal plane, outliers of the tibial and talar components occurred in 11 (13.8%) ankles of each component in the varus deformity group, 3 (7.1%) of each component in the neutral group, and 2 (7.1%) and 1 (3.6%) in the valgus deformity group.

**Table 2:** Accuracy of implant position after total ankle arthroplasty according to preoperative deformity

	Preoperative coronal alignment^a^					Preoperative sagittal alignment^b^			
	Varus (n = 80)	Neutral (n = 42)	Valgus (n = 28)	*p* value		Extension (*n* = 85)	Neutral (*n* = 58)	Flexion (n = 7)	*p* value
α angle^a^	92.4 ± 2.7	91.6 ± 2.4	90.7 ± 2.8	0.061	β angle^a^	84.5 ± 3.8	84.6 ± 2.8	83.8 ± 1.3	0.808
Outcomes^b^					Outcomes^b^				
Excellent	41 (51.2%)	30 (71.5%)	15 (53.6%)	0.244	Excellent	52 (61.2%)	41 (70.7%)	5 (71.4%)	0.769
Acceptable	28 (35.0%)	9 (21.4%)	11 (39.3%)		Acceptable	23 (27.0%)	12 (20.7%)	2 (28.6%)	
Outlier	11 (13.8%)	3 (7.1%)	2 (7.1%)		Outlier	10 (11.8%)	5 (8.6%)	–	
γ angle^a^	91.9 ± 3.3	91.2 ± 2.9	90.2 ± 2.6	0.286	δ angle^a^	91.4 ± 3.9	91.3 ± 4.2	90.4 ± 6.0	0.225
Outcomes^b^					Outcomes^b^				
Excellent	43 (53.8%)	29 (69.1%)	18 (64.3%)	0.385	Excellent	45 (52.9%)	29 (50.0%)	2 (28.6%)	0.584
Acceptable	26 (32.4%)	10 (23.8%)	9 (32.1%)		Acceptable	25 (29.4%)	18 (31.0%)	2 (28.6%)	
Outlier	11 (13.8%)	3 (7.1%)	1 (3.6%)		Outlier	15 (17.7%)	11 (19.0%)	3 (42.8%)	

^a^Values are given as the mean and the standard deviation

^b^Values are given as the number of ankles with the percentage in parentheses

Neutral is within 5° of tibiotalar angle on anteroposterior radiograph. Varus and valgus ankles are defined as those greater than 5° from optimal values

Neutral is within 5° of extension and flexion on lateral radiograph. Extension and flexion ankles are defined as those greater than 5° from optimal values

The ideal value of α, γ, and δ angle is 90°, that of β angle is 84°. Outcome was defined as “excellent” when values were within 3°, “acceptable” when within 5°, and as “outlier” when more than 5° from optimum value. Outcome values are shown as percentage with the number of ankles in parentheses

As done with coronal plane analysis, we evaluated the accuracy of implant position depending on preoperative sagittal deformity: extension, neutral, and flexion. In sagittal plane, outliers of the tibial and talar components occurred in 10 (11.8%) and 15 (17.7%) ankles in the extension group, 5 (8.6%) and 11 (19.0%) in the neutral group, and 0 (0.0%) and 3 (42.8%) in the flexion group, respectively. However, there were no meaningful differences between the 3 groups in the accuracy of component position according to preoperative deformity in coronal and sagittal plane.

In addition, presence of joint incongruency did not increase the outlier rate (Table 3).

**Table 3:** Accuracy of implant position after total ankle arthroplasty according to preoperative ankle congruency

	Varus (*n* = 80)			Valgus (*n* = 28)		
	Congruent (*n* = 46)	Incongruent (*n* = 34)	*p* value	Congruent (*n* = 18)	Incongruent (*n* = 10)	*p* value
α angle^a^	92.2 ± 3.0	92.8 ± 2.3	0.327	90.7 ± 2.9	90.8 ± 2.8	0.915
Outcomes^b^						
Excellent	23 (50.0%)	18 (52.9%)	0.906	10 (55.6%)	5 (50.0%)	0.999
Acceptable	16 (34.8%)	12 (35.3%)		7 (38.9%)	4 (40.0%)	
Outlier	7 (15.2%)	4 (11.8%)		1 (5.5%)	1 (10.0%)	
γ angle^a^	91.7 ± 3.5	92.1 ± 3.0	0.540	90.7 ± 2.5	89.2 ± 2.5	0.156
Outcomes^b^						
Excellent	26 (56.5%)	17 (50.0%)	0.278	11 (61.1%)	7 (70.0%)	0.379
Acceptable	12 (26.1%)	14 (41.2%)		7 (38.9%)	2 (20.0%)	
Outlier	8 (17.4%)	3 (8.8%)		–	1 (10.0%)	

^a^Values are given as the mean and the standard deviation

^b^Value are given as the number of ankles with the percentage parentheses

α and γ angles are measured on anteroposterior radiographs between the longitudinal axis of the tibia and the articulating surface of the tibial component or talar component

β and δ angles are measured on lateral radiographs between the longitudinal axis of the tibia and the articulating surface of the tibial component or talar component

The ideal values of α, γ and δ angles are 90°, that of β angle is 84°. Outcomes were defined as “excellent” when values were within 3°, “acceptable” when within 5°, and as “outlier” when more than 5° from optimum values

## Discussion

The most important finding of the present study is that the outlier rate of component was higher than we expected, and the outlier rate of each component in coronal plane is about 10%, but the talar component position in sagittal plane showed the outlier rate up to 20%. Our results highlight a common problem in TAA, which is the lack of repeatability in implantation position, particularly the talar component. Malalignment of component following TAA has been reported in about 0 to 35% of patients, even though components were thought to be appropriately placed at the time of the operation [12, 30]. There are several reports concerning the current radiographic outcomes of TAA [5, 6, 24, 31–34]. Mann et al. [24] analyzed the outcomes of TAA in 84 ankles. The average overall alignment of the tibial component was 3.0° of varus. Fifty seven (78%) ankles had varus tilt averaging 4.6°, and 10 (14%) ankles had valgus tilt of the talar component averaging 2.8°. Nine (10.7%) outliers occurred in which the tibial component was placed in more than 10° of extension and exceeded 5° of varus. However, Rippstein et al. [6] reported relatively good outcomes after TAA in 240 Mobility ankles. Ninety-three percent of components were correctly centered in the coronal plane, and 97.4% were correctly centered in the sagittal plane. The tibial components were placed on average 2.1 ± 2.9° (−5.5° to 10.2°) in the coronal plane. The mean posterior slope was 6.0 ± 3.8° (−5.8° to 17.1°).

However, there was no comprehensive study concerning the outlier rate of each component or predisposing factors by assessing the accuracy of component after TAA. The present study indicate that outliers rate of the tibial, talar component in coronal plane, and tibial component in sagittal plane showed only about 10.7, 10, and 10%, of cases, but outliers rate of the talar component in sagittal plane occurred in 19.3%. This result suggests that the talar component position intraoperatively is much more difficult than the tibial component position.

There are several factors which can affect alignment of the talar component. Firstly, in operative techniques, the component position in the sagittal plane is a very subjective approach and it concerns different types of ankle prosthesis. In this study, distal tibia cutting block was used as a guide for resection of talar dome. So, the talar component position was affected by distal tibial cutting plane. Such problems may be solved by applying separate talar cutting block without considering the tibia in the process, but this is not easy intraoperatively because there is no distinct landmark and anatomy of talus varies much individually. In addition, stabilizing the foot during talar cutting can only be done manually. Therefore, the talar component position tends to vary and easy to be influenced depending upon foot position, particularly in sagittal alignment. Barg and Lundberg et al. [34, 35] also reported that correct position of the talar component is one of the most technically demanding steps in TAA, which is complicated by changes in the original center of rotation of the tibiotalar joint caused by degeneration and concomitant valgus or varus hindfoot deformities. As a result, sagittal malalignment of the talar component is a common complication of TAA. Novel techniques such as computer-assisted surgery or patient-specific instrumentation could be useful solutions to limit component malalignment.

Based on our results concerning component position according to the preoperative coronal deformity, varus ankles showed a lower rate of excellent outcomes and a higher rate of outlier than neutral or valgus ankles. Previous studies have reported that for varus ankles with ligament imbalance and asymmetric joint loading. Wood and Haskel [5, 36] reported that the failure of TAA in cases of varus deformity was the result of edge loading on the implant caused by ligament instability and remaining ankle malalignment after the operation. Trincat et al. [37] reported that six (29%) ankles underwent revision surgery to correct residual varus malalignment of the hindfoot. Among them, three failures occurred in incongruent ankles (two varus, one valgus) in which the initial procedure did not provide optimal correction of talar tilt. As the degrees of deformity increase, it is difficult to achieve neutral alignment and easy to remain postoperative malalignment. To achieve neutrally aligned ankle after TAA, it is essential to correct ligament imbalance or hindfoot deformity through additional procedures in index surgery. Therefore, precise check of ligament balancing and proper additional procedures are needed, in order to reduce postoperative malalignment.

Our study has some limitations. First, the sample size in neutral and valgus group was relatively small. This may limit our ability to assess outcomes and may evaluate risk factors. Second, we retrospectively reviewed preoperative radiographs, and thus, radiographs were not precisely controlled. It is possible that slight variability of ankle position and rotation influenced assessments the alignment of implant. Finally, this study did not evaluate functional outcomes, which may have been influenced by the component position. Therefore, further studies involving these functional outcomes are required.

## Conclusions

In present study, the outlier rate of components was high as 10 to 20%, especially, the talar component position in sagittal plane showed the outlier rate up to 20% and wider variation. However, presence of preoperative deformity or joint incongruency did not increase the outlier rate of component. Therefore, careful attention should be paid when implanting the talar component in sagittal plane and other reliable instrumentation system is necessary to improve the accuracy of component.

## Abbreviation

TAA: Total ankle arthroplasty

## References

[1] Gougoulias N, Khanna A, Maffulli N (2010). How successful are current ankle replacements?: a systematic review of the literature. Clin Orthop Relat Res.

[2] Alvine FG, Conti SF (2006). The AGILITY ankle: mid- and long-term results. Orthopade.

[3] Hintermann B, Valderrabano V, Dereymaeker G, Dick W (2004). The HINTEGRA ankle: rationale and short-term results of 122 consecutive ankles. Clin Orthop Relat Res.

[4] Lee KT, Lee YK, Young KW, Kim HJ, Park SY, Kim JS (2010). Perioperative complications of the MOBILITY total ankle system: comparison with the HINTEGRA total ankle system. J Orthop Sci.

[5] Wood PL, Deakin S (2003). Total ankle replacement: the results in 200 ankles. J Bone Joint Surg (Br).

[6] Rippstein PF, Huber M, Coetzee JC, Naal FD (2011). Total ankle replacement with use of a new three-component implant. J Bone Joint Surg Am.

[7] Spirt AA, Assal M, Hansen ST (2004). Complications and failure after total ankle arthroplasty. J Bone Joint Surg Am.

[8] Anderson T, Montgomery F, Carlsson A (2003). Uncemented STAR total ankle prostheses. Three to eight-year follow-up of fifty-one consecutive ankles. J Bone Joint Surg Am.

[9] Lee KB, Cho YJ, Park JK, Song EK, Yoon TR, Seon JK (2011). Heterotopic ossification after primary total ankle arthroplasty. J Bone Joint Surg Am.

[10] Primadi A, Xu HX, Yoon TR, Ryu JH, Lee KB (2015). Neurologic injuries after primary total ankle arthroplasty: prevalence and effect on outcomes. J Foot Ankle Res.

[11] Deorio JK, Easley ME (2008). Total ankle arthroplasty. Instr Course Lect.

[12] Doets HC, Brand R, Nelissen RG (2006). Total ankle arthroplasty in inflammatory joint disease with use of two mobile-bearing designs. J Bone Joint Surg Am.

[13] Guyer AJ, Richardson G (2008). Current concepts review: total ankle arthroplasty. Foot Ankle Int.

[14] Knecht SI, Estin M, Callaghan JJ, Zimmerman MB, Alliman KJ, Alvine FG (2004). The Agility total ankle arthroplasty, Seven to sixteen-year follow-up. J Bone Joint Surg Am.

[15] Kofoed H, Lundberg-Jensen A (1999). Ankle arthroplasty in patients younger and older than 50 years: a prospective series with long-term follow-up. Foot Ankle Int.

[16] Trajkovski T, Pinsker E, Cadden A, Daniels T (2013). Outcomes of ankle arthroplasty with preoperative coronal-plane varus deformity of 10° or greater. J Bone Joint Surg Am.

[17] Easley ME, Adams SB, Hembree WC, DeOrio JK (2011). Results of total ankle arthroplasty. J Bone Joint Surg Am.

[18] Fukuda T, Haddad SL, Ren Y, Zhang LQ (2010). Impact of talar component rotation on contact pressure after total ankle arthroplasty: a cadaveric study. Foot Ankle Int.

[19] Henricson A, Skoog A, Carlsson A (2007). The Swedish Ankle Arthroplasty Register: an analysis of 531 arthroplasties between 1993 and 2005. Acta Orthop.

[20] Schutte BG, Louwerens JW (2008). Short-term results of our first 49 Scandinavian total ankle replacements (STAR). Foot Ankle Int.

[21] Skyttä ET, Koivu H, Eskelinen A, Ikävalko M, Paavolainen P, Remes V (2010). Total ankle replacement: a population-based study of 515 cases from the Finnish Arthroplasty Register. Acta Orthop.

[22] Wood PL, Prem H, Sutton C (2008). Total ankle replacement: medium-term results in 200 Scandinavian total ankle replacements. J Bone Joint Surg (Br).

[23] Lee KB, Kim MS, Park KS, Cho KJ, Primadhi A (2013). Effect of anterior translation of the talus on outcomes of three-component total ankle arthroplasty. BMC Musculoskelet Disord.

[24] Mann JA, Mann RA, Horton E (2011). STAR™ ankle: long-term results. Foot Ankle Int.

[25] Jaffe WL, Hawkins CA (1999). Normalized and proportionalized cemented femoral stem survivorship at 15 years. J Arthroplasty.

[26] Ostgaard HC, Helger L, Regnér H, Garellick G (2001). Femoral alignment of the Charnley stem: a randomized trial comparing the original with the new instrumentation in 123 hips. Acta Orthop Scand.

[27] Mullaji AB, Shetty GM, Lingaraju AP, Bhayde S (2013). Which factors increase risk of malalignment of the hip-knee-ankle axis in TKA. Clin Orthop Relat Res.

[28] Ritter MA, Davis KE, Davis P, Farris A, Malinzak RA, Berend ME (2013). Preoperative malalignment increases risk of failure after total knee arthroplasty. J Bone Joint Surg Am.

[29] Heyse TJ, Decking R, Davis J, Boettner F, Laskin RS (2009). Varus gonarthrosis predisposes to varus malalignment in TKA. HSS J.

[30] Pyevich MT, Saltzman CL, Callaghan JJ, Alvine FG (1998). Total ankle arthroplasty: a unique design. Two to twelve-year follow-up. J Bone Joint Surg Am.

[31] Hsu AR, Haddad SL (2015). Early clinical and radiographic outcomes of intramedullary-fixation total ankle arthroplasty. J Bone Joint Surg Am.

[32] Haytmanek CT, Gross C, Easley ME, Nunley JA (2015). Radiographic outcomes of a mobile-bearing total ankle replacement. Foot Ankle Int.

[33] Bonnin M, Gaudot F, Laurent JR, Ellis S, Colombier JA, Judet T (2011). The Salto total ankle arthroplasty: survivorship and analysis of failures at 7 to 11 years. Clin Orthop Relat Res.

[34] Barg A, Elsner A, Anderson AE, Hintermann B (2011). The effect of three-component total ankle replacement malalignment on clinical outcome: pain relief and functional outcome in 317 consecutive patients. J Bone Joint Surg Am.

[35] Lundberg A, Svensson OK, Németh G, Selvik G (1989). The axis of rotation of the ankle joint. J Bone Joint Surg (Br).

[36] Haskell A, Mann RA (2004). Ankle arthroplasty with preoperative coronal plane deformity: short-term results. Clin Orthop Relat Res.

[37] Trincat S, Kouyoumdjian P, Asencio G (2012). Total ankle arthroplasty and coronal plane deformities. Orthop Traumatol Surg Res.

